# Understanding the Physical Nature of Coronal “EIT Waves”

**DOI:** 10.1007/s11207-016-1030-y

**Published:** 2016-12-12

**Authors:** D. M. Long, D. S. Bloomfield, P. F. Chen, C. Downs, P. T. Gallagher, R.-Y. Kwon, K. Vanninathan, A. M. Veronig, A. Vourlidas, B. Vršnak, A. Warmuth, T. Žic

**Affiliations:** 1 Mullard Space Science Laboratory, UCL, Holmbury St. Mary, Dorking, Surrey RH5 6NT UK; 2 School of Physics, Trinity College Dublin, College Green, Dublin 2, Ireland; 3 Northumbria University, Newcastle upon Tyne, NE1 8ST UK; 4 School of Astronomy & Space Science, Nanjing University, 163 Xianlin Ave., Nanjing, 210023 P.R. China; 5 Predictive Science Inc., 9990 Mesa Rim Rd., Suite 170, San Diego, CA 92121 USA; 6 College of Science, George Mason University, 4400 University Drive, Fairfax, VA 22030 USA; 7 Kanzelhöhe Observatory/IGAM, Institute of Physics, University of Graz, 8010 Graz, Austria; 8 Applied Physics Laboratory, The Johns Hopkins University, Laurel, MD 20723 USA; 9 Faculty of Geodesy, Hvar Observatory, Kaciceva 26, 10000 Zagreb, Croatia; 10 Leibniz-Institut für Astrophysik Potsdam (AIP), An der Sternwarte 16, 14482 Potsdam, Germany

**Keywords:** Coronal mass ejections, low coronal signatures, Waves, magnetohydrodynamic, Waves, propagation, Waves, shock

## Abstract

For almost 20 years the physical nature of globally propagating waves in the solar corona (commonly called “EIT waves”) has been controversial and subject to debate. Additional theories have been proposed over the years to explain observations that did not agree with the originally proposed fast-mode wave interpretation. However, the incompatibility of observations made using the *Extreme-ultraviolet Imaging Telescope* (EIT) onboard the *Solar and Heliospheric Observatory* with the fast-mode wave interpretation was challenged by differing viewpoints from the twin *Solar Terrestrial Relations Observatory* spacecraft and data with higher spatial and temporal resolution from the *Solar Dynamics Observatory*. In this article, we reexamine the theories proposed to explain EIT waves to identify measurable properties and behaviours that can be compared to current and future observations. Most of us conclude that the so-called EIT waves are best described as fast-mode large-amplitude waves or shocks that are initially driven by the impulsive expansion of an erupting coronal mass ejection in the low corona.

## Introduction

Globally propagating waves in the solar corona have been studied in detail since they were first directly observed by the *Extreme-ultraviolet Imaging Telescope* (EIT: Delaboudinière *et al.*, 1995) onboard the *Solar and Heliospheric Observatory* (SOHO: Domingo, Fleck, and Poland, 1995). However, a physical explanation for “EIT waves” (as they are commonly called) has remained elusive due to a paucity of observations and inconsistent analyses. This has led to the continued development of competing theories designed to explain the phenomenon.

EIT waves are generally observed as bright pulses in the low solar corona emanating from the source of a solar eruption, and often traverse the solar disk in less than an hour. They can have velocities of up to ${\approx\,}1400~\mbox{km}\,\mbox{s}^{-1}$ (*cf.* Nitta *et al.*, 2013), but are most typically observed at velocities of $200\,\mbox{--}\,500~\mbox{km}\,\mbox{s}^{-1}$ (Klassen *et al.*, 2000; Thompson and Myers, 2009; Muhr *et al.*, 2014). It was initially suggested that they were magnetohydrodynamic (MHD) fast-mode waves driven either by the erupting coronal mass ejection (CME) or alternatively by the associated flare (*e.g.* Moses *et al.*, 1997; Dere *et al.*, 1997; Thompson *et al.*, 1998). This was consistent with the global MHD fast-mode wave propagating in the corona that was predicted by Uchida (1968) to explain the chromospheric Moreton–Ramsey wave (Moreton, 1960; Moreton and Ramsey, 1960).

However, EIT wave velocities were found to be much lower than estimated quiet-Sun coronal fast-mode speeds, leading some to suggest that they could not be fast-mode waves. While EIT waves exhibit the wave attributes of reflection (*e.g.* Gopalswamy *et al.*, 2009), refraction (*e.g.* Wills-Davey and Thompson, 1999), and transmission (*e.g.* Olmedo *et al.*, 2012), they can remain stationary at coronal hole (CH) boundaries for tens of minutes to hours (*e.g.* Delannée, 2000) – behaviour originally proposed as inconsistent with the wave interpretation.

These discrepancies led to the development of several alternative explanations for EIT waves. One branch elaborated on the wave interpretation, treating EIT waves as slow-mode waves (*cf.* Wang, Shen, and Lin, 2009), slow-mode solitons (*e.g.* Wills-Davey, DeForest, and Stenflo, 2007), or more generally as shock waves (or large-amplitude MHD waves, for more details see the review by Vršnak and Cliver, 2008). The other branch eschewed waves entirely, instead treating them as pseudo-waves resulting from coronal magnetic-field reconfiguration during CME eruption. In this approach, EIT-wave brightenings result from several different processes, including stretching of magnetic-field lines (*cf.* Chen *et al.*, 2002), Joule heating in a current shell (*cf.* Delannée, Hochedez, and Aulanier, 2007), or continuous small-scale reconnection (*cf.* Attrill *et al.*, 2007).

As with other aspects of solar eruptive events, EIT waves are a relatively common phenomenon. Although they are less common than CMEs (every EIT wave has an associated CME, but the converse is not necessarily true; Biesecker *et al.*, 2002), between 1997 and 2013 at least 407 events have been identified using the SOHO, *Solar Terrestrial Relations Observatory* (STEREO: Kaiser *et al.*, 2008), and *Solar Dynamics Observatory* (SDO: Pesnell, Thompson, and Chamberlin, 2012) spacecraft (*cf.* Thompson and Myers, 2009; Nitta *et al.*, 2013; Muhr *et al.*, 2014). Despite this, EIT waves tend to be studied in isolation, using single-event studies to make generalised statements about their physical interpretation. This approach led to a disconnect between advocates of the wave and pseudo-wave interpretations, with both sides using different (and in most cases single-event) observations to support their preferred view.

The majority of the theories designed to explain this phenomenon were originally proposed based on observations from SOHO/EIT that had a spatial and temporal sampling of ${\approx\,}5^{\prime\prime}$ and 12 – 15 minutes, respectively. This typically provided two observations of an EIT wave per event, and it places restrictions on the resulting physical interpretation. However, this was improved on by the *Extreme UltraViolet Imager* (EUVI: Wuelser *et al.*, 2004) onboard the twin STEREO spacecraft (${\approx\,}3.2^{\prime\prime}$ and 1.25 – 10 minutes) and more recently the *Atmospheric Imaging Assembly* (AIA: Lemen *et al.*, 2012) onboard SDO (${\approx\,}1.2^{\prime\prime}$ and 12 seconds). Although this improvement in both spatial and temporal resolution should allow a more rigorous testing of all of the different interpretations for EIT waves, this has not been the case, primarily because very few testable predictions of physical properties and behaviour are provided for each proposed theory.

This article should be viewed as being complementary to the reviews of EIT waves by Wills-Davey and Attrill (2009), Gallagher and Long (2011), Zhukov (2011), Patsourakos and Vourlidas (2012), Liu and Ofman (2014), and in particular the recent reviews by Warmuth (2015) and Chen (2016). This is achieved by identifying all of the currently measurable properties (and some beyond our current capabilities) for each of the theories or models. A direct comparison is then performed for each of these properties, making use of the most recently published results (*e.g.* observations of differential emission measure and simulations that relate EIT waves to other solar phenomena). This effort is a result of an International Working Team on “The Nature of Coronal Bright Fronts” convened at the International Space Science Institute (ISSI: www.issibern.ch).

In this article, we aim to identify and quantify the physical properties and behaviour predicted by the theories that have been proposed to explain the EIT-wave phenomenon, greatly expanding on the initial attempt by Patsourakos *et al.* (2009). Each theory is outlined in Section 2 with particular emphasis placed on what they each predict for a variety of physical properties, including kinematics, height, bounded area, and variation in density, temperature, and magnetic-field strength. The analysis techniques currently used to identify and study EIT waves and the limitations that they naturally impose on the observations are outlined in Section 3, allowing the optimal technique for each property to be identified. Finally, the best interpretation for EIT waves given current analysis techniques is summarised in Section 4.

## Theories

The development of multiple theories to explain EIT waves can be primarily attributed to inconsistencies in interpretation. This was compounded by the small number of events studied in detail by multiple authors, including (but not limited to) the events from 12 May 1997 (*e.g.* Moses *et al.*, 1997; Dere *et al.*, 1997; Thompson *et al.*, 1998), 19 May 2007 (*e.g.* Long *et al.*, 2008; Veronig, Temmer, and Vršnak, 2008; Gopalswamy *et al.*, 2009; Attrill, 2010), 13 February 2009 (*e.g.* Patsourakos and Vourlidas, 2009; Cohen *et al.*, 2009; Kienreich, Temmer, and Veronig, 2009), and 15 February 2011 (*e.g.* Schrijver *et al.*, 2011; Olmedo *et al.*, 2012; Vanninathan *et al.*, 2015). This approach (initially driven by the relatively small number of well-observed events in the SOHO/EIT era) led to a situation where the theories proposed to explain EIT waves were primarily developed to explain the behaviour of individual and necessarily different events, while paying minimal attention to predicting more generalised behaviour and observables that may help to understand the true nature of these waves. The launches of STEREO and SDO have led to more statistical EIT-wave studies using the analysis techniques developed for individual events, but these have focused on individual properties such as kinematics and wave-pulse characteristics (*e.g.* Thompson and Myers, 2009; Warmuth and Mann, 2011; Nitta *et al.*, 2013; Muhr *et al.*, 2014).

There are two main branches of proposed theories: wave (Table 1, columns 2 – 4), and pseudo-wave (Table 1, columns 5 – 7). In the wave interpretation, EIT waves are classified using the MHD wave equations as linear fast-mode waves (*e.g.* Thompson *et al.*, 1998), or alternatively as nonlinear waves such as large-amplitude fast-mode or shock waves (*e.g.* Vršnak and Cliver, 2008) or MHD slow-mode solitons (*cf.* Wills-Davey, DeForest, and Stenflo, 2007). In the pseudo-wave interpretation, the waves are described as brightenings arising from magnetic-field-line stretching (Chen *et al.*, 2002), Joule heating in current shells (Delannée, Hochedez, and Aulanier, 2007; Delannée *et al.*, 2008), or continuous small-scale reconnection (Attrill *et al.*, 2007). The physical processes demanded by these different interpretations should result in different observed behaviour, thus providing an opportunity to distinguish between theories.

**Table 1:** Prediction of physical properties of pulses from theory.

Pulse physical property	Wave theories			Pseudo-wave theories/models		
	Fast-mode		Slow-mode soliton	Field-line stretching^a^	Current shell	Continuous reconnection
	Small amp. linear wave	Large amp. wave/shock				
Phase velocity [*v*]	$v^{\mathrm{f}}_{\mathrm{p}}$	${>\,}v^{\mathrm{f}}_{\mathrm{p}}$; ∝ *U* ^b^	∝ *U* ^b^	${<\,}v^{\mathrm{f}}_{\mathrm{p}}$	$v_{\mathrm{CME}_{\perp}}$	$v_{\mathrm{CME}_{\perp}}$
	${>\,}v_{\mathrm{CME}_{\perp}}$	${>\,}v_{\mathrm{CME}_{\perp}}$	…	$v_{\mathrm{CME}_{\perp}}$	…	…
Acceleration [*a*]	0	< 0	0	$a_{\mathrm{CME}_{\perp}}$	$a_{\mathrm{CME}_{\perp}}$	$a_{\mathrm{CME}_{\perp}}$
Broadening	≈ 0	> 0	0	> 0	$f(a_{\mathrm{CME}_{\perp}})$	$f(v_{\mathrm{CME}_{\perp}}, t_{\mathrm{cooling}})$
Δ*B*	≳ 0	> 0	< 0	> 0	≈ 0	< 0
Δ*T*	Adia.	Adia. + *Q*	Adia.	Adia.	$Q_{\mathrm{Joule}}$ + adia.	Non-adia.
$\Delta n_{\mathrm{e}}$	Compression	Compression	Compression	Compression	Compression	Upflows
Height	$f(B, n_{\mathrm{e}})$	$f(B, n_{\mathrm{e}})$	$f(B, n_{\mathrm{e}})$	*f* (CME bubble)	≈ 280 or 407 Mm^c^	${<\,}10~\mbox{Mm}$ ^d^
Area bounded	${>\,}A_{\mathrm{CME}}$	${>\,}A_{\mathrm{CME}}$	${>\,}A_{\mathrm{CME}}$	$A_{\mathrm{CME}}$	$A_{\mathrm{CME}}$	$A_{\mathrm{CME}}$
Rotation	Possible	Possible	Possible	Possible	Possible	Possible
Reflection	Yes	Yes	Possible	No	No	No
Refraction	Yes	Yes	Possible	No	No	No
Transmission	Yes	Yes	Yes	No	No	No
Stationary fronts	Yes	Yes	No	Yes	Yes	Yes
Co-spatial Type II	No	Possible	No	No	No	No
Moreton wave	No	Possible	No	No	No	Possible

^a^Describing only the slower component of the two-wave scenario (*i.e.* the density perturbation component).

^b^ $U=I_{\mathrm{peak}}/I_{0}$ (*i.e.* the ratio of peak intensity [$I_{\mathrm{peak}}$] to background intensity [$I_{0}$]).

^c^Delannée *et al.* (2008).

^d^Height of adjacent small-scale loops; value quoted in Patsourakos *et al.* (2009).

However, a detailed discussion of properties and behaviour predicted by each theory was often omitted in their initial presentation, with the result that conclusions about their validity continue to be made based on observations of single properties (*e.g.* kinematics or pulse characteristics). In this section, we attempt to overcome this issue and identify observable properties predicted by each of the different theories and models proposed to explain the EIT-wave phenomenon. The predicted properties for each are presented in Table 1 under the assumption of an idealised homogeneous background corona, while Sections 2.1–2.5 outline the individual theories and the reasoning behind their physical predictions.

### MHD Fast-Mode Waves

**Figure 1:**
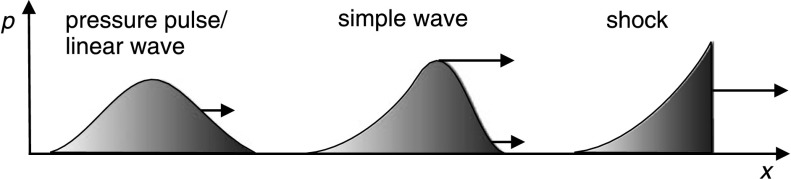
Graphical representation of the wave models, highlighting the differences between pulse wave forms (adapted from Figure 2 of Warmuth, 2007). The linear slow-mode and fast-mode waves involve small perturbations and so take the pulse form in the left panel. Nonlinear effects become more important as the amplitude increases, with the pulse taking the form of a simple wave in the centre panel. A special solution of the nonlinear wave equations can involve this steepening being canceled out by dispersive effects, leading to the formation of an MHD soliton. Alternatively, the simple wave may become shocked, taking the pulse form in the right panel.

The MHD wave equations may be solved to produce two linearised magnetoacoustic wave types (with forms depicted in Figure 1, left panel), namely slow- and fast-mode waves (*e.g.* Priest, 2014). For both, the phase velocity [$v_{\mathrm{p}}^{\mathrm{s}}$ and $v^{\mathrm{f}}_{\mathrm{p}}$, respectively] is defined by the sound speed [$c_{\mathrm{s}}$] and the Alfvén velocity [$v_{\mathrm{A}}$] of the medium through which they propagate (*i.e.* the corona), but also by the angle between the direction of propagation and that of the magnetic field. Slow-mode waves have velocities of $0~\mbox{km}\,\mbox{s}^{-1}$ perpendicular to the magnetic field, while for fast-mode waves it is dependent on the temperature, density, and magnetic field in the corona. EIT waves travel across the Sun where the field is primarily radial, so they cannot be interpreted as slow-mode waves.

In addition to linear forms of the MHD fast-mode wave, EIT waves can also be interpreted as large-amplitude pulses using nonlinear wave theory as discussed by Warmuth (2007), Vršnak and Cliver (2008), and Lulić *et al.* (2013). With this approach, if the amplitude of the wave is sufficiently large, the nonlinear terms become important and the crest of the wave can move faster than the characteristic speed of the medium through which it is passing. This so-called simple wave (*cf.* Mann, 1995a) therefore begins to steepen (Figure 1, centre panel), and may ultimately form a shock wave (Figure 1, right panel). This interpretation of EIT waves as global shock waves was initially motivated by their strong association with metric Type II radio bursts that indicate the presence of a shock front (*e.g.* Klassen *et al.*, 2000; Biesecker *et al.*, 2002; Vršnak and Cliver, 2008).

The most simple definition of a shock wave is of a discontinuity travelling faster than the characteristic speed of the ambient medium through which it is propagating; in this case, the fast-mode velocity of the solar corona. However, it is possible for a piston-driven shock (wherein the motion of a piston drives a shock that the medium cannot flow around) to be formed even if the piston has a velocity lower than the characteristic speed of the medium. In this situation, it is also possible for the shock to travel faster than and therefore decouple from the piston before propagating freely. Interpretation as a shock front raised discussion as to the initial driver, with opposing camps claiming that the EIT wave was initially driven either by the rapid release of energy during the impulsive phase of a flare (*cf.* Vršnak, 2001), or alternatively by the rapid expansion of a CME in the low corona (*e.g.* Cliver *et al.*, 2005). However, both camps agreed that once formed, the EIT wave would then decouple from its driver and become a freely propagating shocked simple wave (*cf.* Landau and Lifshitz, 1959) that decelerates, consistent with the piston-driven shock interpretation.

As well as the velocity and acceleration of the pulse, variations in pulse width can be compared to that predicted by theory. Both of the fast-mode wave types introduced above exhibit broadening during propagation. For small- and large-amplitude waves this may result from superposition of multiple frequencies within the pulse (*i.e.* dispersion), while wave steepening may also contribute to broadening for large-amplitude waves or shocks. In terms of physical properties, an increase in magnetic-field strength [$B$], should be produced during the passage of both fast-mode types (Priest, 1982), but it will be negligible for small-amplitude waves. Small- and large-amplitude waves both produce temperature and density increases, which should be non-adiabatic for shocks and adiabatic otherwise.

Geometrically, the vertical extent of both wave types should be dependent on how the density and magnetic field vary with height above the Sun. As both are initially driven by the erupting CME, their spatial extent at any point in time should match or exceed the spatial extent of the associated CME. The wave front could appear to rotate during the driven phase if the CME driver is elliptical and itself rotates. As these are true wave solutions, they are expected to behave as such, undergoing reflection and refraction where appropriate. A direct consequence of this is the transmission of a portion of the wave front through a CH and apparent stationary wave fronts at the CH boundary.

In terms of additional phenomena associated with fast-mode waves, only the large-amplitude wave or shock provides the necessary conditions to produce co-spatial Type II radio bursts (through the presence of a shock) and Moreton–Ramsey waves (given sufficient pressure acting downwards on the chromosphere).

### MHD Slow-Mode Solitons

The concept of the EIT wave as an MHD slow-mode soliton was proposed by Wills-Davey, DeForest, and Stenflo (2007) in an attempt to explain some of the discrepancies between the observed properties of EIT waves and predictions of linear MHD fast-mode wave theory. In particular, the authors identified several issues where predictions did not match observations, namely the value and variety of observed pulse velocities and what this means for the theoretical assumption of a low-$\beta$ plasma in the corona and the coherence of the pulse over the duration of its observation. It was argued that these issues made the MHD fast-mode wave interpretation unfeasible, instead suggesting that they were most consistent with the interpretation of the pulse as an MHD slow-mode soliton.

Although Wills-Davey, DeForest, and Stenflo (2007) only give a brief qualitative argument for solitons as a candidate mechanism for EIT waves, we can estimate some of their physical properties here. The MHD slow-mode soliton provides a special solution to the nonlinear MHD wave equations, with the nonlinear steepening of the wave (Figure 1, centre panel) exactly canceled out by dispersive effects. This allows a wave packet (*i.e.* soliton) to form that is observed as a bright pulse propagating at constant velocity (*i.e.* $a=0$) and width (*i.e.* no broadening). The velocity is dependent on the amplitude of the pulse intensity, $U=I_{\mathrm{peak}}/I_{0}$ (*i.e.* the ratio of peak intensity [$I_{\mathrm{peak}}$] to background intensity [$I_{0}$]), indicating that brighter, higher-amplitude pulses will exhibit greater velocities.

As with the fast-mode waves in Section 2.1, the MHD slow-mode soliton should result in an adiabatic increase in both temperature and density, although this is accompanied by a decrease in magnetic-field strength (since the gas and magnetic pressures are out of phase for slow-mode waves). Similarly, the vertical extent of the soliton will be a function of the background magnetic-field strength and density, while the lateral extent will exceed that of the associated CME. In addition, an MHD slow-mode soliton should exhibit rotation with propagation given a rotating elliptical driver, and may undergo reflection and refraction under specific conditions. MHD slow-mode solitons do not interact with CH boundaries in the same manner as fast-mode waves or shocks, resulting in their transmission through CHs without producing stationary fronts.

The MHD slow-mode soliton is not a shock and so does not produce the necessary conditions for either a co-spatial Type II radio burst or a chromospheric Moreton-Ramsey wave, similar to the small-amplitude fast-mode wave.

### Field-Line Stretching Model

The field-line stretching model was originally proposed by Chen *et al.* (2002) to reconcile observations indicating no clear relationship between solar flares and EIT waves (*e.g.* Delannée and Aulanier, 1999) and simulations of EIT waves using unrealistic plasma-$\beta$ values (*cf.* Wu *et al.*, 2001). In order to overcome these issues, Chen *et al.* (2002) performed a 2D simulation using the time-dependent compressible resistive MHD equations solved using a multi-step implicit scheme (Hu, 1989; Chen and Shibata, 2000). This was expanded on by Chen, Fang, and Shibata (2005), where two different field configurations were used to study the effect of neighbouring active regions. The model of Chen *et al.* (2002) and Chen, Fang, and Shibata (2005) predicts two propagating features in a CME eruption: a fast-mode shock wave (termed “coronal Moreton wave”), and a slower density perturbation resulting from the stretching of field lines overlying the erupting flux rope (termed EIT wave). The fast-mode shock wave is covered in Section 2.1, while the slower density perturbation is discussed here.

**Figure 2:**
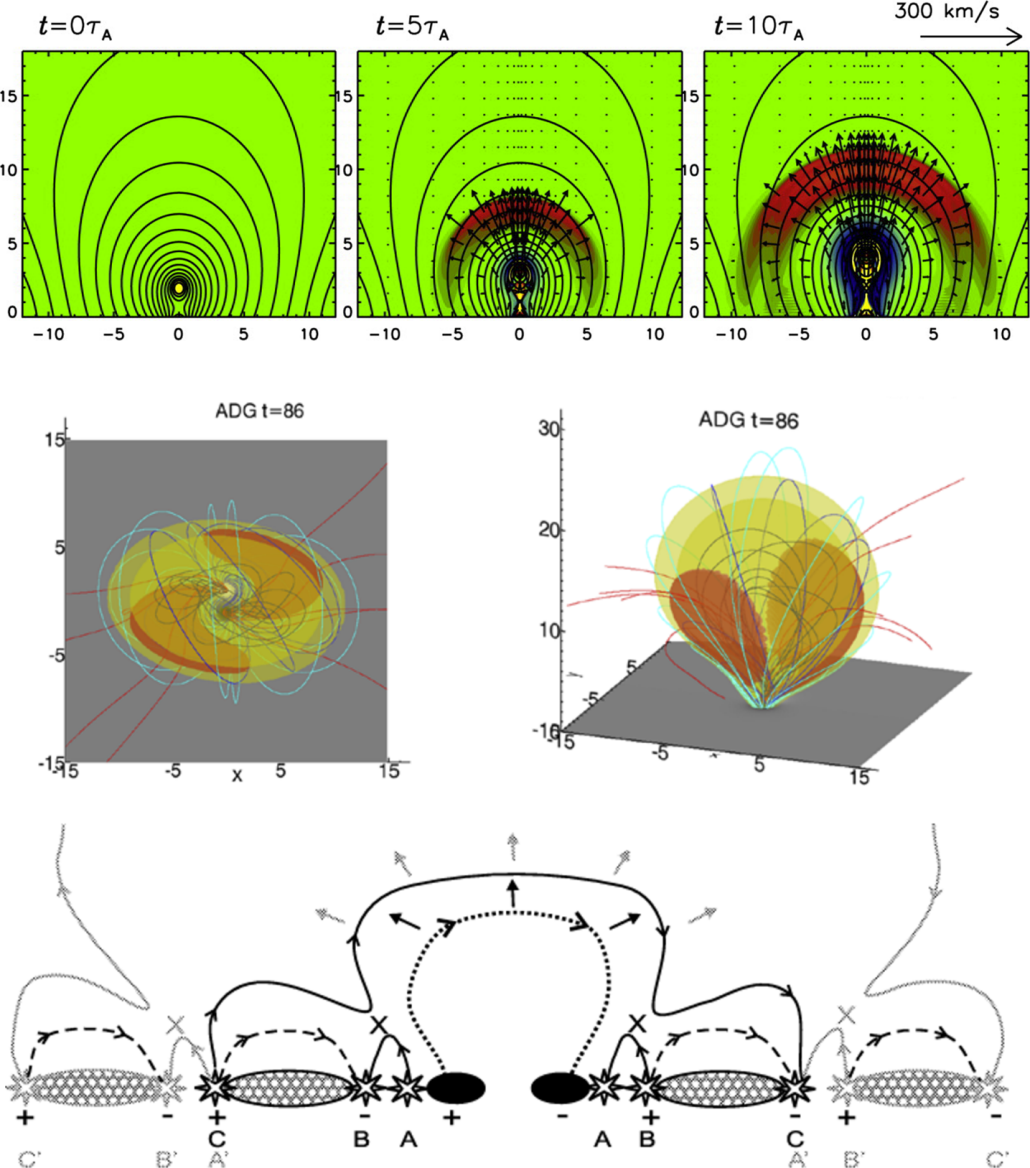
Graphical representations of the pseudo-wave theories and models: field-line stretching model (top row, modified from Figure 1 of Chen *et al.*, 2002); current-shell model (middle row, modified from Figure 2 of Delannée *et al.*, 2008); continuous reconnection (bottom row, reproduced from Figure 4 of Attrill *et al.*, 2007).

With an erupting CME driving the stretching of field lines (Figure 2, top row), the lateral velocity of the density perturbation is constrained by the geometry of the overlying magnetic field. Under the assumption of a semicircular overlying field (as adopted by Chen *et al.*, 2002), the density perturbation should have a lateral velocity ${\approx\,}1/3$ that of the associated fast-mode wave. However, it is noted that this should be considered an upper limit because the lateral velocity will be lower when field lines are radially elongated rather than being semicircular. The time taken for information on the stretching of the overlying field to reach the low corona increases as successively higher field lines are perturbed. Outside the source active region, this yields decreasing lateral velocity (*i.e.* $a<0$) for the low-corona density perturbation (*i.e.* at the foot-points of the overlying field).

The stretching of overlying magnetic-field lines manifests itself as an increase in magnetic-field strength, with the density increasing due to the frozen-in effect. Numerical simulations performed by Chen, Fang, and Shibata (2005) also suggest a weak temperature increase in the density perturbation. The successive outward stretching of field lines has a component in all directions (*i.e.* not only vertically), resulting in a broadening of the pulse in time (Chen and Shibata, 2002).

Chen, Fang, and Shibata (2005) noted that the dimming region within the CME bubble (characterised by strong upflows) should define the area enclosed by the density perturbation. This model also predicts a dome-like structure for the density perturbation, constrained by the height of the driving CME (Chen, 2009). Rotation of the density perturbation is not present in 2D simulations, but it is expected in 3D when the background magnetic field is sheared. The density perturbation is not expected to show reflection or refraction at CH boundaries, instead possibly resulting in long-lived stationary brightenings at CH edges.

The stretching of magnetic-field lines does not produce a shock, and hence no co-spatial Type II radio burst is expected. In addition, the density perturbation is not expected to be energetically capable of producing a Moreton–Ramsey wave. However, the coronal fast-mode shock also produced in this model was proposed to be able to produce both of these phenomena if it is of sufficient amplitude.

### Current-Shell Model

The current-shell model proposed by Delannée, Hochedez, and Aulanier (2007) aimed to explain the non-isotropic nature of EIT waves that they suggested was incompatible with an MHD fast-mode wave. The model also aimed to explain previous observations of long-duration stationary bright features associated with EIT waves (Delannée, 2000). In all three events studied by Delannée (2000), part of the observed bright feature propagates while another part remains stationary from frame to frame, a behaviour that these authors had suggested to be inconsistent with the interpretation of the disturbance as a wave.

Instead, Delannée, Hochedez, and Aulanier (2007) proposed that as the coronal magnetic field opens during the eruption of a CME, electric currents should form as a result of sudden jumps in magnetic-field connectivity. As shown in the middle row of Figure 2, this produces a large-scale current shell that separates an erupting flux rope from the surrounding coronal field. The current shell dissipates its energy via Joule heating, resulting in an EUV brightening at these locations. As a result, the kinematics of the wave will be consistent with the lateral expansion of the erupting CME bubble (*i.e.* $v_{\mathrm{CME}_{\perp}}$ and $a_{\mathrm{CME}_{\perp}}$). Similarly, both the height and area of the pulse will be related to the height and area of the CME bubble. The measured width of the pulse will strongly depend on the lateral velocity of the CME and the plasma cooling time. However, the 3D nature of a current shell means there will be an additional contribution as the emission from several heights is combined along the line of sight. The temporal evolution of all of these components determines whether or not the pulse is observed to broaden in time.

Although an increase in density is not required to produce the Joule heating (and hence increased temperature), the expanding nature of the CME leads to a density enhancement through compression at the leading edge of the CME bubble. The physical processes involved also suggest that it is unlikely to exhibit any clear variation in magnetic-field strength associated with the observed disturbance.

Rotation of the propagating disturbance was seen in simulations by Delannée *et al.* (2008), but no reflection or refraction is expected at CH boundaries. The combination of the current shell with the open-field topology of CHs leads to deflection of the CME bubble or sustained reconnection at the CH boundary for unfavourable or favourable magnetic-field orientation, respectively. Neither of these scenarios results in transmission through the CH, while the latter can produce a long-lived intensity enhancement (*i.e.* stationary front) at the boundary.

As with field-line stretching in Section 2.3, Joule heating itself will not produce a Type II burst or a Moreton–Ramsey wave, as no shock is formed.

### Continuous Reconnection

The EIT-wave concept of continuous reconnection was originally proposed by Attrill *et al.* (2007) to explain two of their observed properties: the rotation of several EIT waves with propagation, and the appearance of remote coronal-dimming regions associated with erupting CMEs. Two events from 7 April 1997 and 12 May 1997 were chosen to illustrate these effects, as both were very clear EIT waves that appeared to rotate with propagation (an observation originally noted by Podladchikova and Berghmans, 2005). It was found that the direction of rotation exhibited by the EIT waves was defined by the helicity of the erupting active region. Although it had been suggested that EIT waves could be blast waves driven by the energy release in a solar flare, Attrill *et al.* (2007) suggested that the observed rotation was incompatible with this interpretation. Instead, they proposed that EIT waves were due to reconnection between the magnetic structure of the erupting CME and the surrounding coronal field.

In the continuous reconnection model, an EIT-wave brightening is the result of systematic reconnection between the erupting CME flux rope and surrounding (favourably oriented) loops and open field lines in the quiet Sun. As these field lines reconnect, a steady stream of particles is accelerated at the reconnection sites toward the chromosphere (as in solar flares, but on much smaller and weaker scales). A series of brightenings will be created via chromospheric evaporation as these particles impact the dense lower atmosphere, with brightenings observed as an EIT wave (Figure 2, bottom row). As a result, this should exhibit signatures consistent with those observed during a flare (*i.e.* strongly increased temperatures and densities due to upflowing hot material), albeit on a smaller scale. In addition, there may be minor variations in the magnetic-field strength.

The reconnection process between the erupting CME and the adjacent small-scale coronal loops means that the kinematics of the pulse should be defined by the lateral motion of the erupting CME (*i.e.* $v_{\mathrm{CME}_{\perp}}$ and $a_{\mathrm{CME}_{\perp}}$). The combination of this lateral motion and the plasma cooling timescale [$t_{\mathrm {cooling}}$] define the apparent width of the pulse and its temporal variation. In addition, the area bounded by the pulse is defined by the lateral extent of the CME, with the pulse being formed low in the solar atmosphere at heights comparable to quiet-Sun loops. As with the current-shell model, the EIT wave produced by continuous reconnection is not expected to exhibit reflection or refraction at CH boundaries, but it can result in long-lived stationary bright fronts through interchange reconnection (*e.g.* Attrill *et al.*, 2006).

Although the low-coronal nature of the reconnection process suggests that it may be possible for Moreton–Ramsey waves to be produced, this will depend upon the amount of energy released during reconnection. However, there will be no signature of a Type II radio burst as no shock is formed in this approach.

## Data Analysis and Modelling

As summarised in Table 1, the theories proposed to explain EIT waves make separate predictions for physical properties, each of which may be measured and used to confirm the interpretation. However, the techniques used to observe and analyse EIT waves can influence the value and behaviour of the different properties being measured. EIT waves are traditionally observed as broad and diffuse low-intensity features that are difficult to identify in single intensity images, and as a result, they are often identified using movies or difference images (where a leading image is subtracted from a following image). The temporal step used when subtracting images can affect the size, shape, and derived velocity of the pulse, therefore care must be taken when using difference images. However, the advent of multiple passbands for observing EIT waves and improvements in image processing and analysis techniques are providing an opportunity to simultaneously study multiple properties of these features, allowing a better distinction of the theories.

### Current Observational and Analysis Capabilities

The kinematics of observed EIT waves are the easiest property to measure, and as a result they have been calculated since EIT waves were first observed. The multitude of proposed theories arose primarily from discrepancies between the observed and predicted behaviour of the pulse kinematics, and it continues to be the primary method of differentiating between theories. However, it is clear from Table 1 that kinematics alone are not sufficient to discriminate between theories and other properties should be taken into account. It has been suggested that estimates of kinematics can be strongly affected by observational temporal cadence (*e.g.* Long *et al.*, 2008; Byrne *et al.*, 2013), although Muhr *et al.* (2014) found no relationship between observing cadence and pulse velocity.

As previously noted, EIT waves are difficult to identify in single images, often requiring movies to enable identification. Extensive image processing is therefore required to allow the identification of EIT waves. This is primarily achieved using difference or ratio images, where an image has a preceding image subtracted from or divided into it, respectively. These allow a feature to be identified by highlighting changes in intensity as the position of the feature changes between the two images. Although the temporal step between images does not affect the identification of the EIT-wave leading edge, it can affect the observed pulse properties (*e.g.* width and amplitude). For example, short temporal steps suppress long-term intensity variations that are due to the motion of the background corona and highlight fast-moving features. Conversely, long temporal steps highlight long-term variations that become progressively less clear as the temporal step increases.

The optimal temporal step for estimating kinematics and properties of an EIT wave must therefore be chosen carefully. The temporal step must be large enough that the positions of the pulse do not overlap in the two images, as this leads to incorrect estimation of pulse properties such as width and peak intensity difference (or intensity ratio). Similarly, the temporal step must be sufficiently small that the pulse can be identified above the noise of the varying background corona. This approach allows an estimate to be made of the variation in pulse position and (depending on the temporal step) the width of the pulse with time.

After identifying a pulse, several methods can be used to study it. The position can be identified manually in a series of images using a point-and-click approach (*e.g.* Narukage and Shibata, 2004; White and Thompson, 2005; Thompson and Myers, 2009), but this method is user-dependent and subject to bias. Recent work has tended to use automated methods that minimise user involvement and employ predefined properties of the pulse to identify and track it. Although automation makes them relatively self-consistent, algorithms such as CorPITA (Long *et al.*, 2014) and Solar Demon (Kraaikamp and Verbeeck, 2015, previously NEMO; Podladchikova and Berghmans, 2005) may not necessarily return the same values for pulse properties as those identified manually. In addition, Huygens tracking was proposed to interpolate between pulse positions (Wills-Davey, 2006), but to the best of the authors’ knowledge, this approach has not been used elsewhere.

Both CorPITA and Solar Demon measure the kinematics of the pulse along defined directions using intensity profiles, a technique also often used for manual estimates of pulse kinematics. This approach collapses the intensity along an arc into a 1D intensity plot (*e.g.* Muhr *et al.*, 2011; Long *et al.*, 2014), allowing the pulse to be identified as an increase in intensity that may be fitted and tracked using a predefined model. An alternative approach is a stack plot that combines 1D profiles into a 2D image (*e.g.* Liu *et al.*, 2010; Ma *et al.*, 2011; Shen and Liu, 2012b), allowing the pulse to be visually identified. Both approaches have merit: 1D spatial profiles allow the amplitude, width, or shape of a pulse to be tracked and studied; stack plots allow the identification of additional fronts that may result from projection effects as a CME erupts. Stack plots have also been shown to be useful when looking for reflection and refraction of wave pulses at CH and active-region boundaries (*e.g.* Gopalswamy *et al.*, 2009; Olmedo *et al.*, 2012; Kienreich *et al.*, 2013), while slicing along the temporal axis yields a temporal profile from one location, enabling studies of pulse passage effects on the background corona.

The volume of data and improved spatial and temporal resolution now available from current and upcoming instrumentation provides an opportunity for improved discrimination between different theories. It is now possible to visualise heating and cooling in the pulse using the subtly different temperature responses of the different SDO/AIA passbands. As described by Downs *et al.* (2012), the 171 Å, 193 Å, and 211 Å passbands may be combined and used to indicate changes in the temperature of the plasma during the passage of an EIT wave. This may be used as an additional constraint on the interpretation of the feature.

A more detailed analysis to quantify the amount of heating or cooling requires a full estimation of the differential emission measure (DEM) of the plasma. Although this requires knowledge of a series of emission lines obtained from spectroscopy, recent advances in analysis techniques and the temperature ranges covered by the multiple EUV passbands of SDO/AIA are beginning to allow the calculation of DEMs using broadband images. Kozarev *et al.* (2011) used the DEM analysis of a limb eruption on 13 June 2011 to find a density increase of 12 %, while Vanninathan *et al.* (2015) used a similar approach for an on-disk event on 15 February 2011. Using the regularized-inversion technique of Hannah and Kontar (2012, 2013), they found a density increase of 6 – 9 %, corresponding to a temperature increase of ${\approx\,}5\,\mbox{--}\,6~\%$ during the EIT-wave passage, which was shown to be a result of adiabatic compression at the wave front.

Although this approach provides an estimate of the variation in temperature and density associated with the EIT wave, it is restricted by the broadband nature of the instrument, which sacrifices spectral resolution for spatial resolution. The alternative to this is provided by slit spectrometers such as the *Extreme-ultraviolet Imaging Spectrometer* (EIS: Culhane *et al.*, 2007) onboard the *Hinode* spacecraft (Kosugi *et al.*, 2007). EIS has very high spectral resolution from a $1\,\mbox{--}\,2^{\prime\prime}$-wide slit that provides an opportunity to identify up- and down-flows associated with the passage of the pulse. However, the very small field of view of EIS and the anisotropic nature of EIT waves make it difficult to observe a pulse, and spectrometric observations of EIT waves remain extremely rare. Despite this, EIS has observed at least two events, both of which have been extensively studied (*cf.* Harra *et al.*, 2011; Veronig *et al.*, 2011; Chen *et al.*, 2011; Long *et al.*, 2013).

The EIT waves studied using DEM techniques and observed by *Hinode*/EIS exhibited signatures consistent with a shock wave interpretation, including rapid temperature and density increases and very high velocities. However, the primary signatures used to indicate the presence of a shock wave are Type II radio bursts (*e.g.* Klein *et al.*, 1999; Mann, 1995b; Mann and Classen, 1995). Dynamic radio spectra are sufficient to identify the existence of a shock wave and potentially its height (inferred from a density model or estimate), but they do not identify the spatial location of the shock. However, this can be occasionally achieved using radio-imaging observations from, *e.g.*, the Nançay Radioheliograph. This facility was used by Pohjolainen *et al.* (2001), Khan and Aurass (2002), Vršnak *et al.* (2005, 2006), and Carley *et al.* (2013) to identify and track Type II radio emission associated with EIT waves, complemented by dynamic spectra from multiple instruments to study the associated shock-accelerated particle emission. Unfortunately, radio imaging of Type II bursts and CMEs is exceptionally rare because we lack dedicated solar facilities around the world, while Type II emission is a sufficient but not necessary condition for the existence of a shock.

Although chosen as the primary discriminant between different theories and models of EIT waves, the kinematics of the pulse cannot and should not be considered as conclusive proof that the feature conforms to one interpretation at the expense of all others. The interaction of the pulse with the surrounding corona is also of vital importance, with signatures of reflection and/or refraction providing an opportunity to immediately discriminate between wave and pseudo-wave interpretations. Any study of EIT waves should also include the variation of temperature and density of the plasma associated with the propagation of the pulse and radio observations of a shock, or lack thereof. A combination of current instruments provides multiple discriminants that may be used to add to the weight of evidence in favour of one theory or another. In addition, there may be other properties or observations beyond our current capability (*e.g.* changes in coronal magnetic field) that could be used to definitively identify the physical processes involved in the development and propagation of an EIT wave.

### Modelling and Simulations

An additional tool that may be used to understand and interpret EIT waves, and indeed potentially discriminate between theories, is modelling of the eruption and evolution of the EIT wave itself. This is not a new approach, with many of the original theories designed to explain the phenomenon proposed following simulations of solar eruptions (*e.g.* Delannée, 2000; Chen *et al.*, 2002). The complexity and realism of the simulations and modelling vary widely, with both simple analytical modelling and more complex numerical modelling, including 3D MHD models providing different insights into the processes involved.

Most of the analytical modelling has focused on characterising how the large-amplitude wave front forms as a result of the explosive expansion of a three-dimensional piston. This approach has been studied in detail by many groups, with Vršnak and Lulić (2000a,b) in particular describing how this process evolves. More recent work has focused on the details of the formation mechanism, the timescales over which the wave front forms, and the exact nature of the original piston (*e.g.* Žic *et al.*, 2008; Temmer *et al.*, 2009; Lulić *et al.*, 2013).

An alternative approach is to model the formation and evolution of the EIT wave numerically. As described by Vršnak *et al.* (2016), this can be done in one of two ways: either using realistic configurations for the initial eruption and the background corona to study specific events (*e.g.* Cohen *et al.*, 2009; Schmidt and Ofman, 2010; Downs *et al.*, 2011, 2012), or alternatively using a simplified configuration to understand the general processes involved (*e.g.* Chen *et al.*, 2002; Chen, Fang, and Shibata, 2005; Wang, Shen, and Lin, 2009; Hoilijoki *et al.*, 2013; Wang *et al.*, 2015). Both approaches offer unique insights into the initiation and evolution of the pulse and the effects of the background corona.

Simulations and modelling provide a unique opportunity to examine the behaviour of EIT waves and compare the observations with that predicted by theory. In particular, combining simulations with the different observations offered by instruments such as SDO/AIA, *Hinode*/EIS, and the twin viewpoints of the STEREO spacecraft offers a powerful new tool to examine this phenomenon.

### Future Diagnostics

While current instrumentation and techniques provide an unprecedented view of the Sun, particularly the low corona, some gaps in observations remain. This is especially true of the coronal magnetic field, with Table 1 identifying different behaviours for the theories and models (notably providing discrimination in both the wave and pseudo-wave categories). Current magnetic-field observations use Stokes polarization measurements and the Zeeman effect to estimate field strength and orientation in the photosphere where the signal is strong. However, low signal-to-noise ratio in coronal emission lines means that rather than measuring the coronal field directly, the photospheric field must be extrapolated to infer the coronal field, particularly outside active regions, because the magnetic field is relatively weak.

Some advances have been made in measuring the coronal magnetic field – *e.g.* the *Coronal Multi-channel Polarimeter* (CoMP: Tomczyk *et al.*, 2008), which uses the Fe xiii 10747 Å emission line to measure the Stokes polarization in the low corona at heights of ${\approx\,}1.03\,\mbox{--}\,1.5~\mbox{R}_{\odot}$. However, the signal-to-noise ratio for the CoMP is quite low and, combined with the ground-based nature of the instrument, this makes observations of EIT waves with the CoMP extremely challenging. One of the aims of the forthcoming *Daniel K. Inouye Solar Telescope* (DKIST) is to estimate the coronal magnetic field, with its four-meter primary mirror greatly improving the signal-to-noise ratio. As a result, it may be possible to measure variations in coronal magnetic-field strength during the passage of an EIT wave, enabling the $\Delta B$ row in Table 2 to be addressed. However, the small field of view for DKIST (*i.e.* ${<\,}100^{\prime\prime}$) means that EIT-wave observations will most likely be serendipitous.

**Table 2:** Observational support for model or theory predictions: agreement (✓); not inconsistent (∼); no measurements available (–); no agreement (×).

Pulse physical property	Wave theories			Pseudo-wave theories/models		
	Fast-mode		Slow-mode soliton	Field-line stretching^a^	Current shell	Continuous reconnection
	Small amp. linear wave	Large amp. wave/shock				
Phase velocity [*v*]	✓	✓	∼	∼	×	×
Acceleration [*a*]	✓	✓	✓	×	×	×
Broadening	✓	✓	✓	✓	–	–
Δ*B*	–	–	–	–	–	–
Δ*T*	✓	✓	✓	✓	✓	×
$\Delta n_{\mathrm{e}}$	✓	✓	✓	✓	✓	×
Height	✓	✓	✓	✓	×	×
Area bounded	✓	✓	✓	×	×	×
Rotation	∼	∼	∼	✓	✓	✓
Reflection	✓	✓	✓	×	×	×
Refraction	✓	✓	✓	×	×	×
Transmission	✓	✓	✓	×	×	×
Stationary fronts	✓	✓	×	✓	✓	✓
Co-spatial Type II	×	✓	×	×	×	×
Moreton wave	×	✓	×	×	×	✓

^a^Describing only the slower component of the two-wave scenario (*i.e.* the density perturbation component).

## Discussion

The range of theories proposed to explain EIT waves (outlined in Section 2) and the number of articles and reviews devoted to this phenomenon show that they remain a subject of interest to the broader community. However, it is also clear that many of the original interpretations were affected by the relatively low temporal and spatial resolutions of SOHO/EIT. Multi-point observations from the STEREO spacecraft provide better insight into the relation between EIT waves and CMEs, while the improved temporal and spatial capabilities of SDO/AIA could supply sufficient evidence of the physical processes at work.

Table 1 was constructed to indicate the different properties and behaviours that may be used to distinguish between the different theories. How each of these predictions compares to observations is presented in Table 2 with symbols indicating that observations and predictions are in agreement (✓), observations are not inconsistent with predictions (∼), measurements have not been or cannot yet be made (–), and observations do not match predictions (×). The properties given in Tables 1 and 2 can be grouped into five main headings, each of which is discussed in more detail below: kinematic properties, physical properties, geometric properties, spatio-temporal properties, and associated phenomena.

### Kinematic Properties

As the easiest property to identify for a global pulse, the velocity [$v$] predicted for each theory is clearly defined in Table 1. As fast-mode waves, the small- and large-amplitude waves should propagate at or somewhat above the fast-mode wave velocity, respectively. Statistical studies of EIT waves using SOHO/EIT and STEREO/EUVI found average velocities of $200\,\mbox{--}\,500~\mbox{km}\,\mbox{s}^{-1}$ (*cf.* Klassen *et al.*, 2000; Thompson and Myers, 2009; Muhr *et al.*, 2014), consistent with fast-mode waves. Although recent studies using SDO/AIA report higher average velocities of $600\,\mbox{--}\,730~\mbox{km}\,\mbox{s}^{-1}$ (Nitta *et al.*, 2013; Liu and Ofman, 2014), it should be noted that these consider the maximum velocity in any direction rather than directional averages like earlier works. While some studies indicate that higher-intensity pulses have greater velocities (Muhr *et al.*, 2014), pulse intensity may be affected by the background corona (Nitta *et al.*, 2013), making comparison between pulse amplitude and velocity difficult. Hence, observations are not inconsistent with the MHD slow-mode soliton. Existing studies of lateral CME expansion (*e.g.* Patsourakos, Vourlidas, and Stenborg, 2010) clearly show the formation and decoupling of the EIT wave from the CME driver, behaviour inconsistent with the predictions made by the MHD slow-mode soliton and pseudo-wave models.

All of the wave theories predict pulse accelerations [$a$] that are either lower than zero or equal to zero, matching results found by many authors (*e.g.* Warmuth *et al.*, 2004a; Long, DeLuca, and Gallagher, 2011; Zheng *et al.*, 2012; Nitta *et al.*, 2013). Distinct correlations are found between EIT-wave initial velocities and acceleration – faster events show stronger deceleration, while those near quiet-Sun coronal fast-mode speeds have no significant deceleration (Warmuth and Mann, 2011; Muhr *et al.*, 2014) – a relation expected for large-amplitude fast-mode waves. In contrast, the field-line stretching, current-shell, and continuous reconnection models predict bright-front acceleration equal to the lateral acceleration of the expanding CME. The observed process of EIT waves decoupling from the CME, as discussed above, requires a difference in lateral acceleration that is inconsistent with the pseudo-wave models.

### Physical Properties

Broadening (or not) of the pulse with propagation is well defined for the wave and field-line stretching models. MHD slow-mode solitons and small-amplitude fast-mode waves predict a pulse with minimal or no broadening, while the large-amplitude fast-mode wave and field-line stretching models predict clear pulse broadening with propagation. This behaviour has been reported many times (*e.g.* Warmuth *et al.*, 2004b; Long *et al.*, 2011; Muhr *et al.*, 2011), supporting the wave and field-line stretching models. Although they do not require a pulse to broaden with propagation, such behaviour is not inconsistent with the current-shell and continuous-reconnection models. For these, broadening depends on the lateral acceleration of the CME (current-shell model) or the lateral velocity of the CME and cooling time of the plasma (continuous reconnection model).

Each theory predicts that EIT waves will increase in temperature and density, although the mechanisms producing this increase differ. The wave, field-line stretching, and current-shell models yield density increases from compression, while for continuous reconnection it is upflowing chromospheric plasma. Compression is clearly observed in EIT waves, supporting the wave, field-line stretching, and current-shell models (*cf.* Kozarev *et al.*, 2011; Ma *et al.*, 2011). Spectroscopic observations show downflows of up to $20~\mbox{km}\,\mbox{s}^{-1}$ at the EIT-wave front (Harra *et al.*, 2011; Veronig *et al.*, 2011), indicative of downward plasma motion related to compression and inconsistent with continuous reconnection.

Recently Schrijver *et al.* (2011) and Vanninathan *et al.* (2015) have shown that temperature enhancements in EIT waves are due to adiabatic heating, consistent with the wave, field-line stretching, and partially also the current-shell model. This is inconsistent with the continuous-reconnection model that predicts an increase due to non-adiabatic processes (*i.e.* low-energy magnetic reconnection). The temperature increase reported by Vanninathan *et al.* (2015) was highest at the peak and frontal part of the EIT wave. While such behaviour is expected for a compressive fast-mode wave and the field-line stretching model, the current-shell model predicts the largest increase in the rear part of the EIT wave. The temperature enhancement is an adiabatic process in the fast-mode wave and field-line stretching models, such that the largest temperature increase occurs with the largest density increase (*i.e.* cospatial with the EUV wave). In the current-shell model, the EIT wave pulse is explained by Joule heating in the current shell that builds up at the separation layer between the erupting flux rope and the surrounding field. Thus, the temperature increase is expected to be highest at this CME-wave interface (*i.e.* in the rear portion of the EUV wave).

Although each of the models makes a distinct prediction for the variation in magnetic field across the EIT-wave pulse, this property cannot be measured using current observational techniques. As noted in Section 3.3, measuring the magnetic field in the corona is hampered by the very low signal-to-noise ratio of magnetically sensitive coronal lines. Detection of a small perturbation in the magnetic field during the passage of a fast, diffuse wave is therefore beyond the capabilities of current instrumentation, and thus these predictions cannot currently be tested.

### Geometric Properties

The wave theories all suggest that the height over which the wave is observed should vary as a function of the magnetic field and density of the background corona, while the area bounded by the pulse should exceed the area bounded by the associated CME. In contrast, the pseudo-wave theories predict an EIT wave with a height defined by the CME leading loop (field-line stretching model) at fairly constant heights of ${\approx\,}280$ or 407 Mm (current-shell model; values from Delannée *et al.*, 2008), or at heights ${<\,}10~\mbox{Mm}$ (continuous reconnection model; value from Patsourakos *et al.*, 2009). In addition, the area bounded by the EIT wave should equal the area of the CME in all pseudo-wave cases.

The visibility of a 3D wave perturbation in a gravitationally stratified corona is expected to be weighted by density (which controls emissivity), with the density scale height being ${\approx\,}70\,\mbox{--}\,90~\mbox{Mm}$ for typical quiet-Sun temperatures of ${\approx\,}1.0\,\mbox{--}\,1.7~\mbox{MK}$. These heights are roughly consistent with the observed heights of EIT waves measured using intensity-based diagnostics (*cf.* Patsourakos and Vourlidas, 2009; Patsourakos *et al.*, 2009; Kienreich, Temmer, and Veronig, 2009) that are significantly different from the predictions of the current-shell and continuous-reconnection models. Observations of dome-shaped EIT waves (*e.g.* Veronig *et al.*, 2010) and dome-shaped fronts connecting to features in the extended corona (*e.g.* Cheng *et al.*, 2012; Kwon, Zhang, and Olmedo, 2014) are consistent with the wave expectations of a 3D perturbation being present at larger heights. In addition, after decoupling, the area bounded by an EIT wave exceeds that of the associated CME (*e.g.* Patsourakos and Vourlidas, 2009) and coronal-dimming region (*e.g.* Veronig *et al.*, 2010), supporting the wave-theory predictions.

### Spatio-Temporal Properties

Spatio-temporal properties of EIT waves feature prominently in predictions of the different theories, which is expected given that they are moving features. The most obvious are reflection, refraction, and transmission of the pulse when faced with an active region or CH boundary. These properties provide the starkest contrasts between the wave and pseudo-wave branches, with wave theories all predicting that EIT waves can show reflection and refraction and are expected to transmit, while no pseudo-wave theories predict these behaviours. However, observations provide clear evidence of reflection (*e.g.* Gopalswamy *et al.*, 2009; Kumar and Manoharan, 2013), refraction (*e.g.* Ofman and Thompson, 2002; Shen and Liu, 2012a), and transmission (*e.g.* Olmedo *et al.*, 2012; Shen *et al.*, 2013).

Interaction between EIT waves and CH boundaries leads to observations of stationary bright fronts at the edges of CHs. This was initially attributed to being due to reconnection between the erupting CME and magnetic field in the CH (*e.g.* Delannée, 2000; Delannée, Hochedez, and Aulanier, 2007; Attrill *et al.*, 2007). Recently, Kwon *et al.* (2013) reported on observations of stationary fronts at CH boundaries in the low corona while the associated EIT wave continued to propagate in the higher corona through magnetic streamers. This behaviour is supported by simulations of wave pulses interacting with sudden changes in density and magnetic field that can produce stationary bright features at the interface region (B. Vršnak, in preparation, 2017). Although MHD slow-mode solitons by definition should not show any variation that is due to a sudden change in density and/or magnetic field, it may be possible for a small- or large-amplitude fast-mode wave to produce a short-lived, stationary bright feature.

Observations that were first reported by Podladchikova and Berghmans (2005) and subsequently by Attrill *et al.* (2007, 2014) have suggested that some EIT waves exhibit rotation during their propagation. This is most consistent with the eruption of a rotating CME, and it matches predictions made by the field-line stretching, current-shell, and continuous-reconnection models. However, it could also be explained by the rotation of an erupting elliptical CME that drives a wave pulse that subsequently propagates freely. As a result, rotation (or lack thereof) is not inconsistent with the wave interpretations.

### Associated Phenomena

As well as the CME associated with the propagating EIT wave, the proposed theories must account for co-spatial Type II radio emission and Moreton–Ramsey waves. The only theory that can definitely produce Type II radio emission that is co-spatial with an EIT wave is that of a large-amplitude wave/shock, as the generation of a Type II burst requires the formation of a shock. This has been confirmed by multiple observations (*e.g.* Pohjolainen *et al.*, 2001; Khan and Aurass, 2002; Vršnak *et al.*, 2005, 2006; Carley *et al.*, 2013) providing evidence of a co-spatial Type II radio burst that track the propagating EIT wave.

Although observations of Moreton–Ramsey waves are rare, they always have associated and co-spatial EIT waves with kinematics consistent between the two phenomena (*e.g.* Warmuth *et al.*, 2001; Veronig *et al.*, 2006; Muhr *et al.*, 2010; Asai *et al.*, 2012). The large-amplitude wave or shock interpretation can generate a Moreton–Ramsey wave, as it can have sufficient energy to compress the upper chromosphere and produce the required “down–up” plasma motions observed in H$\upalpha$ spectra (*cf.* Dodson and Hedeman, 1964; Vršnak *et al.*, 2016). Simultaneous H$\upalpha$, EUV, and radio imaging observations have confirmed the co-occurrence of these phenomena (*e.g.* Thompson *et al.*, 2000; White and Thompson, 2005). Although the field-line stretching and current shell that occur during the eruption of a CME cannot produce a Moreton–Ramsey wave, the process of continuous reconnection could produce small-scale chromospheric brightenings that might be interpreted as a Moreton–Ramsey wave.

## Conclusions

We have identified 15 fundamental EIT-wave properties that may be used to distinguish between the different theories proposed to explain the waves. These are outlined in Table 1 and include kinematic, physical, geometric, and spatio-temporal properties of EIT waves and their associated phenomena. The properties have been characterised using the original articles that proposed the theories and a detailed investigation of the physics underpinning each interpretation. Although this list may not be exhaustive, we believe this table provides all of the necessary information to distinguish between interpretations and should be used when determining the nature of the global wave pulse being studied.

The techniques employed to measure these properties can also have significant impact on their accuracy. Different analysis techniques are optimised for studying different properties, and care is needed to ensure the most appropriate techniques are used. In addition, it may not be possible to measure some properties using the available techniques. For example, the coronal magnetic-field strength is currently very difficult to measure outside active regions, forcing assumptions to be made about the pulse in order to estimate the magnetic-field strength *via* seismological techniques (*e.g.* Mann *et al.*, 1999; Warmuth and Mann, 2005; Long *et al.*, 2013). Although it is important for understanding the structure of the quiet solar corona and how that can affect the directionality of eruptions (*cf.* Möstl *et al.*, 2015), it precludes using observed changes in field strength to diagnose the nature of the pulse. Moreover, while trends in temperature and density can be estimated via imaging in multiple EUV passbands (*e.g.* SDO/AIA), precise measurements require spectroscopy, which is hampered by the small fields of view that are available to slit instruments.

Despite these limitations, Table 2 shows how the predictions of the proposed theories stand up to existing observations. Although it is currently not possible to test some of the predictions, or some properties have not yet been measured, the vast majority have been tested. Given the content of Table 2, most of the authors conclude that propagating EIT-wave pulses are most consistent with the fast-mode large-amplitude wave or shock interpretation, while P.F. Chen insists that two types of EUV wave should be distinguished, and only the faster component can be described as a fast-mode wave (Chen, 2016). While there may be stretching of field lines during the CME eruption and/or formation of a current shell with associated Joule heating and/or reconnection between the erupting CME and the surrounding corona, all of these processes would apply to the CME bubble rather than the pulse that is occasionally observed to propagate ahead of it.

## References

[CR1] Asai A., Ishii T.T., Isobe H., Kitai R., Ichimoto K., UeNo S. (2012). First simultaneous observation of an H$\upalpha$ Moreton wave, EUV wave, and filament/prominence oscillations. Astrophys. J. Lett..

[CR2] Attrill G.D.R. (2010). Dispelling illusions of reflection: A new analysis of the 2007 May 19 coronal “Wave” event. Astrophys. J..

[CR3] Attrill G., Nakwacki M.S., Harra L.K., van Driel-Gesztelyi L., Mandrini C.H., Dasso S., Wang J. (2006). Using the evolution of coronal dimming regions to probe the global magnetic field topology. Solar Phys..

[CR4] Attrill G.D.R., Harra L.K., van Driel-Gesztelyi L., Démoulin P. (2007). Coronal “Wave”: Magnetic footprint of a coronal mass ejection?. Astrophys. J. Lett..

[CR5] Attrill G.D.R., Long D.M., Green L.M., Harra L.K., van Driel-Gesztelyi L. (2014). Extreme-ultraviolet observations of global coronal wave rotation. Astrophys. J..

[CR6] Biesecker D.A., Myers D.C., Thompson B.J., Hammer D.M., Vourlidas A. (2002). Solar phenomena associated with “EIT Waves”. Astrophys. J..

[CR7] Byrne J.P., Long D.M., Gallagher P.T., Bloomfield D.S., Maloney S.A., McAteer R.T.J. (2013). Improved methods for determining the kinematics of coronal mass ejections and coronal waves. Astron. Astrophys..

[CR8] Carley E.P., Long D.M., Byrne J.P., Zucca P., Bloomfield D.S., McCauley J., Gallagher P.T. (2013). Quasiperiodic acceleration of electrons by a plasmoid-driven shock in the solar atmosphere. Nat. Phys..

[CR9] Chen P.F. (2009). The relation between EIT waves and coronal mass ejections. Astrophys. J. Lett..

[CR10] Chen P.F. (2016). Global coronal waves. Low-Frequency Waves in Space Plasmas.

[CR11] Chen P.F., Fang C., Shibata K. (2005). A full view of EIT waves. Astrophys. J..

[CR12] Chen P.F., Shibata K. (2000). An emerging flux trigger mechanism for coronal mass ejections. Astrophys. J..

[CR13] Chen P.F., Shibata K., Ikeuchi S., Hearnshaw J., Hanawa T. (2002). A further consideration of the mechanism for EIT waves. 8th Asian-Pacific Regional Meeting.

[CR14] Chen P.F., Wu S.T., Shibata K., Fang C. (2002). Evidence of EIT and Moreton waves in numerical simulations. Astrophys. J. Lett..

[CR15] Chen F., Ding M.D., Chen P.F., Harra L.K. (2011). Spectroscopic analysis of interaction between an extreme-ultraviolet imaging telescope wave and a coronal upflow region. Astrophys. J..

[CR16] Cheng X., Zhang J., Olmedo O., Vourlidas A., Ding M.D., Liu Y. (2012). Investigation of the formation and separation of an extreme-ultraviolet wave from the expansion of a coronal mass ejection. Astrophys. J. Lett..

[CR17] Cliver E.W., Laurenza M., Storini M., Thompson B.J. (2005). On the origins of solar EIT waves. Astrophys. J..

[CR18] Cohen O., Attrill G.D.R., Manchester W.B., Wills-Davey M.J. (2009). Numerical simulation of an EUV coronal wave based on the 2009 February 13 CME event observed by STEREO. Astrophys. J..

[CR19] Culhane J.L., Harra L.K., James A.M., Al-Janabi K., Bradley L.J., Chaudry R.A. (2007). The EUV imaging spectrometer for Hinode. Solar Phys..

[CR20] Delaboudinière J.-P., Artzner G.E., Brunaud J., Gabriel A.H., Hochedez J.F., Millier F. (1995). EIT: Extreme-ultraviolet imaging telescope for the SOHO mission. Solar Phys..

[CR21] Delannée C. (2000). Another view of the EIT wave phenomenon. Astrophys. J..

[CR22] Delannée C., Aulanier G. (1999). CME associated with transequatorial loops and a bald patch flare. Solar Phys..

[CR23] Delannée C., Hochedez J.-F., Aulanier G. (2007). Stationary parts of an EIT and Moreton wave: A topological model. Astron. Astrophys..

[CR24] Delannée C., Török T., Aulanier G., Hochedez J.-F. (2008). A new model for propagating parts of EIT waves: A current shell in a CME. Solar Phys..

[CR25] Dere K.P., Brueckner G.E., Howard R.A., Koomen M.J., Korendyke C.M., Kreplin R.W. (1997). EIT and LASCO observations of the initiation of a coronal mass ejection. Solar Phys..

[CR26] Dodson H.W., Hedeman E.R. (1964). Moving material accompanying the flare of 1959 July $16^{d}21^{h}14^{m}~\mbox{UT}$. NASA Spec. Publ..

[CR27] Domingo V., Fleck B., Poland A.I. (1995). The SOHO mission: An overview. Solar Phys..

[CR28] Downs C., Roussev I.I., van der Holst B., Lugaz N., Sokolov I.V., Gombosi T.I. (2011). Studying extreme ultraviolet wave transients with a digital laboratory: Direct comparison of extreme ultraviolet wave observations to global magnetohydrodynamic simulations. Astrophys. J..

[CR29] Downs C., Roussev I.I., van der Holst B., Lugaz N., Sokolov I.V. (2012). Understanding SDO/AIA observations of the 2010 June 13 EUV wave event: Direct insight from a global thermodynamic MHD simulation. Astrophys. J..

[CR30] Gallagher P.T., Long D.M. (2011). Large-scale bright fronts in the solar corona: A review of “EIT waves”. Space Sci. Rev..

[CR31] Gopalswamy N., Yashiro S., Temmer M., Davila J., Thompson W.T., Jones S. (2009). EUV wave reflection from a coronal hole. Astrophys. J. Lett..

[CR32] Hannah I.G., Kontar E.P. (2012). Differential emission measures from the regularized inversion of Hinode and SDO data. Astron. Astrophys..

[CR33] Hannah I.G., Kontar E.P. (2013). Multi-thermal dynamics and energetics of a coronal mass ejection in the low solar atmosphere. Astron. Astrophys..

[CR34] Harra L.K., Sterling A.C., Gömöry P., Veronig A. (2011). Spectroscopic observations of a coronal Moreton wave. Astrophys. J. Lett..

[CR35] Hoilijoki S., Pomoell J., Vainio R., Palmroth M., Koskinen H.E.J. (2013). Interpreting solar EUV wave observations from different viewing angles using an MHD model. Solar Phys..

[CR36] Hu Y.Q. (1989). A multistep implicit scheme for time-dependent 2-dimensional magnetohydrodynamic flows. J. Comput. Phys..

[CR37] Kaiser M.L., Kucera T.A., Davila J.M., St. Cyr O.C., Guhathakurta M., Christian E. (2008). The STEREO mission: An introduction. Space Sci. Rev..

[CR38] Khan J.I., Aurass H. (2002). X-ray observations of a large-scale solar coronal shock wave. Astron. Astrophys..

[CR39] Kienreich I.W., Temmer M., Veronig A.M. (2009). STEREO quadrature observations of the three-dimensional structure and driver of a global coronal wave. Astrophys. J. Lett..

[CR40] Kienreich I.W., Muhr N., Veronig A.M., Berghmans D., De Groof A., Temmer M. (2013). Solar TErrestrial Relations Observatory-A (STEREO-A) and PRoject for on-Board Autonomy 2 (PROBA2) quadrature observations of reflections of three EUV waves from a coronal hole. Solar Phys..

[CR41] Klassen A., Aurass H., Mann G., Thompson B.J. (2000). Catalogue of the 1997 SOHO-EIT coronal transient waves and associated type II radio burst spectra. Astron. Astrophys. Suppl..

[CR42] Klein K.-L., Khan J.I., Vilmer N., Delouis J.-M., Aurass H. (1999). X-ray and radio evidence on the origin of a coronal shock wave. Astron. Astrophys..

[CR43] Kosugi T., Matsuzaki K., Sakao T., Shimizu T., Sone Y., Tachikawa S. (2007). The Hinode (solar-B) mission: An overview. Solar Phys..

[CR44] Kozarev K.A., Korreck K.E., Lobzin V.V., Weber M.A., Schwadron N.A. (2011). Off-limb solar coronal wavefronts from SDO/AIA extreme-ultraviolet observations – Implications for particle production. Astrophys. J. Lett..

[CR45] Kraaikamp E., Verbeeck C. (2015). Solar Demon – An approach to detecting flares, dimmings, and EUV waves on SDO/AIA images. J. Space Weather Space Clim..

[CR46] Kumar P., Manoharan P.K. (2013). Eruption of a plasma blob, associated M-class flare, and large-scale extreme-ultraviolet wave observed by SDO. Astron. Astrophys..

[CR47] Kwon R.-Y., Zhang J., Olmedo O. (2014). New insights into the physical nature of coronal mass ejections and associated shock waves within the framework of the three-dimensional structure. Astrophys. J..

[CR48] Kwon R.-Y., Ofman L., Olmedo O., Kramar M., Davila J.M., Thompson B.J., Cho K.-S. (2013). STEREO observations of fast magnetosonic waves in the extended solar corona associated with EIT/EUV waves. Astrophys. J..

[CR49] Landau L.D., Lifshitz E.M. (1959). Fluid Mechanics.

[CR50] Lemen J.R., Title A.M., Akin D.J., Boerner P.F., Chou C., Drake J.F. (2012). The Atmospheric Imaging Assembly (AIA) on the Solar Dynamics Observatory (SDO). Solar Phys..

[CR51] Liu W., Ofman L. (2014). Advances in observing various coronal EUV waves in the SDO era and their seismological applications (invited review). Solar Phys..

[CR52] Liu W., Nitta N.V., Schrijver C.J., Title A.M., Tarbell T.D. (2010). First SDO AIA observations of a global coronal EUV “Wave”: Multiple components and “Ripples”. Astrophys. J. Lett..

[CR53] Long D.M., DeLuca E.E., Gallagher P.T. (2011). The wave properties of coronal bright fronts observed using SDO/AIA. Astrophys. J. Lett..

[CR54] Long D.M., Gallagher P.T., McAteer R.T.J., Bloomfield D.S. (2008). The kinematics of a globally propagating disturbance in the solar corona. Astrophys. J. Lett..

[CR55] Long D.M., Gallagher P.T., McAteer R.T.J., Bloomfield D.S. (2011). Deceleration and dispersion of large-scale coronal bright fronts. Astron. Astrophys..

[CR56] Long D.M., Williams D.R., Régnier S., Harra L.K. (2013). Measuring the magnetic-field strength of the quiet solar corona using “EIT Waves”. Solar Phys..

[CR57] Long D.M., Bloomfield D.S., Gallagher P.T., Pérez-Suárez D. (2014). CorPITA: An automated algorithm for the identification and analysis of coronal “EIT Waves”. Solar Phys..

[CR58] Lulić S., Vršnak B., Žic T., Kienreich I.W., Muhr N., Temmer M., Veronig A.M. (2013). Formation of coronal shock waves. Solar Phys..

[CR59] Ma S., Raymond J.C., Golub L., Lin J., Chen H., Grigis P. (2011). Observations and interpretation of a low coronal shock wave observed in the EUV by the SDO/AIA. Astrophys. J..

[CR60] Mann G. (1995). Simple magnetohydrodynamic waves. J. Plasma Phys..

[CR61] Mann G., Benz A.O., Krüger A. (1995). Theory and observations of coronal shock waves. Coronal Magnetic Energy Releases.

[CR62] Mann G., Classen T. (1995). Shock waves in the solar corona and their radio emission. Adv. Space Res..

[CR63] Mann G., Aurass H., Klassen A., Estel C., Thompson B.J., Vial J.-C., Kaldeich-Schü B. (1999). Coronal transient waves and coronal shock waves. 8th SOHO Workshop: Plasma Dynamics and Diagnostics in the Solar Transition Region and Corona.

[CR64] Moreton G.E. (1960). H$\upalpha$ observations of flare-initiated disturbances with velocities ${\sim\,}1000~\mbox{km/sec}$. Astron. J..

[CR65] Moreton G.E., Ramsey H.E. (1960). Recent observations of dynamical phenomena associated with solar flares. Publ. Astron. Soc. Pac..

[CR66] Moses D., Clette F., Delaboudinière J.-P., Artzner G.E., Bougnet M., Brunaud J. (1997). EIT observations of the extreme ultraviolet Sun. Solar Phys..

[CR67] Möstl C., Rollett T., Frahm R.A., Liu Y.D., Long D.M., Colaninno R.C. (2015). Strong coronal channelling and interplanetary evolution of a solar storm up to Earth and Mars. Nat. Commun..

[CR68] Muhr N., Vršnak B., Temmer M., Veronig A.M., Magdalenić J. (2010). Analysis of a global Moreton wave observed on 2003 October 28. Astrophys. J..

[CR69] Muhr N., Veronig A.M., Kienreich I.W., Temmer M., Vršnak B. (2011). Analysis of characteristic parameters of large-scale coronal waves observed by the solar-terrestrial relations observatory/extreme ultraviolet imager. Astrophys. J..

[CR70] Muhr N., Veronig A.M., Kienreich I.W., Vršnak B., Temmer M., Bein B.M. (2014). Statistical analysis of large-scale EUV waves observed by STEREO/EUVI. Solar Phys..

[CR71] Narukage N., Shibata K., Sakurai T., Sekii T. (2004). Observations of flare-associated waves with SolarB. The Solar-B Mission and the Forefront of Solar Physics.

[CR72] Nitta N.V., Schrijver C.J., Title A.M., Liu W. (2013). Large-scale coronal propagating fronts in solar eruptions as observed by the atmospheric imaging assembly on board the solar dynamics observatory–an ensemble study. Astrophys. J..

[CR73] Ofman L., Thompson B.J. (2002). Interaction of EIT waves with coronal active regions. Astrophys. J..

[CR74] Olmedo O., Vourlidas A., Zhang J., Cheng X. (2012). Secondary waves and/or the “Reflection” from and “Transmission” through a coronal hole of an extreme ultraviolet wave associated with the 2011 February 15 X2.2 flare observed with SDO/AIA and STEREO/EUVI. Astrophys. J..

[CR75] Patsourakos S., Vourlidas A. (2009). “Extreme Ultraviolet Waves” are waves: First quadrature observations of an extreme ultraviolet wave from STEREO. Astrophys. J. Lett..

[CR76] Patsourakos S., Vourlidas A. (2012). On the nature and genesis of EUV waves: A synthesis of observations from SOHO, STEREO, SDO, and Hinode (invited review). Solar Phys..

[CR77] Patsourakos S., Vourlidas A., Stenborg G. (2010). The genesis of an impulsive coronal mass ejection observed at ultra-high cadence by AIA on SDO. Astrophys. J. Lett..

[CR78] Patsourakos S., Vourlidas A., Wang Y.M., Stenborg G., Thernisien A. (2009). What is the nature of EUV waves? First STEREO 3D observations and comparison with theoretical models. Solar Phys..

[CR79] Pesnell W.D., Thompson B.J., Chamberlin P.C. (2012). The Solar Dynamics Observatory (SDO). Solar Phys..

[CR80] Podladchikova O., Berghmans D. (2005). Automated detection of EIT waves and dimmings. Solar Phys..

[CR81] Pohjolainen S., Maia D., Pick M., Vilmer N., Khan J.I., Otruba W. (2001). On-the-disk development of the halo coronal mass ejection on 1998 May 2. Astrophys. J..

[CR82] Priest E.R. (1982). Solar Magneto-Hydrodynamics.

[CR83] Priest E. (2014). Magnetohydrodynamics of the Sun.

[CR84] Schmidt J.M., Ofman L. (2010). Global simulation of an extreme ultraviolet imaging telescope wave. Astrophys. J..

[CR85] Schrijver C.J., Aulanier G., Title A.M., Pariat E., Delannée C. (2011). The 2011 February 15 X2 flare, ribbons, coronal front, and mass ejection: Interpreting the three-dimensional views from the solar dynamics observatory and STEREO guided by magnetohydrodynamic flux-rope modeling. Astrophys. J..

[CR86] Shen Y., Liu Y. (2012). Evidence for the wave nature of an extreme ultraviolet wave observed by the atmospheric imaging assembly on board the solar dynamics observatory. Astrophys. J..

[CR87] Shen Y., Liu Y. (2012). Simultaneous observations of a large-scale wave event in the solar atmosphere: From photosphere to corona. Astrophys. J. Lett..

[CR88] Shen Y., Liu Y., Su J., Li H., Zhao R., Tian Z. (2013). Diffraction, refraction, and reflection of an extreme-ultraviolet wave observed during its interactions with remote active regions. Astrophys. J. Lett..

[CR89] Temmer M., Vršnak B., Žic T., Veronig A.M. (2009). Analytic modeling of the Moreton wave kinematics. Astrophys. J..

[CR90] Thompson B.J., Myers D.C. (2009). A catalog of coronal “EIT Wave” transients. Astrophys. J. Suppl..

[CR91] Thompson B.J., Plunkett S.P., Gurman J.B., Newmark J.S., St. Cyr O.C., Michels D.J. (1998). SOHO/EIT observations of an Earth-directed coronal mass ejection on May 12, 1997. Geophys. Res. Lett..

[CR92] Thompson B.J., Reynolds B., Aurass H., Gopalswamy N., Gurman J.B., Hudson H.S. (2000). Observations of the 24 September 1997 coronal flare waves. Solar Phys..

[CR93] Tomczyk S., Card G.L., Darnell T., Elmore D.F., Lull R., Nelson P.G. (2008). An instrument to measure coronal emission line polarization. Solar Phys..

[CR94] Uchida Y. (1968). Propagation of hydromagnetic disturbances in the solar corona and Moreton’s wave phenomenon. Solar Phys..

[CR95] Vanninathan K., Veronig A.M., Dissauer K., Madjarska M.S., Hannah I.G., Kontar E.P. (2015). Coronal response to an EUV wave from DEM analysis. Astrophys. J..

[CR96] Veronig A.M., Temmer M., Vršnak B. (2008). High-cadence observations of a global coronal wave by STEREO EUVI. Astrophys. J. Lett..

[CR97] Veronig A.M., Temmer M., Vršnak B., Thalmann J.K. (2006). Interaction of a Moreton/EIT wave and a coronal hole. Astrophys. J..

[CR98] Veronig A.M., Muhr N., Kienreich I.W., Temmer M., Vršnak B. (2010). First observations of a dome-shaped large-scale coronal extreme-ultraviolet wave. Astrophys. J. Lett..

[CR99] Veronig A.M., Gömöry P., Kienreich I.W., Muhr N., Vršnak B., Temmer M., Warren H.P. (2011). Plasma diagnostics of an EIT wave observed by Hinode/EIS and SDO/AIA. Astrophys. J. Lett..

[CR100] Vršnak B. (2001). Solar flares and coronal shock waves. J. Geophys. Res..

[CR101] Vršnak B., Cliver E.W. (2008). Origin of coronal shock waves. Invited review. Solar Phys..

[CR102] Vršnak B., Lulić S. (2000). Formation of coronal Mhd shock waves – I. The basic mechanism. Solar Phys..

[CR103] Vršnak B., Lulić S. (2000). Formation of coronal MHD shock waves – II. The pressure pulse mechanism. Solar Phys..

[CR104] Vršnak B., Magdalenić J., Temmer M., Veronig A., Warmuth A., Mann G. (2005). Broadband metric-range radio emission associated with a Moreton/EIT wave. Astrophys. J. Lett..

[CR105] Vršnak B., Warmuth A., Temmer M., Veronig A., Magdalenić J., Hillaris A., Karlický M. (2006). Multi-wavelength study of coronal waves associated with the CME-flare event of 3 November 2003. Astron. Astrophys..

[CR106] Vršnak B., Žic T., Lulić S., Temmer M., Veronig A.M. (2016). Formation of coronal large-amplitude waves and the chromospheric response. Solar Phys..

[CR107] Wang H., Shen C., Lin J. (2009). Numerical experiments of wave-like phenomena caused by the disruption of an unstable magnetic configuration. Astrophys. J..

[CR108] Wang H., Liu S., Gong J., Wu N., Lin J. (2015). Contribution of velocity vortices and fast shock reflection and refraction to the formation of EUV waves in solar eruptions. Astrophys. J..

[CR109] Warmuth A., Klein K.-L., MacKinnon A.L. (2007). Large-scale waves and shocks in the solar corona. The High Energy Solar Corona: Waves, Eruptions, Particles.

[CR110] Warmuth A. (2015). Large-scale globally propagating coronal waves. Living Rev. Solar Phys..

[CR111] Warmuth A., Mann G. (2005). A model of the Alfvén speed in the solar corona. Astron. Astrophys..

[CR112] Warmuth A., Mann G. (2011). Kinematical evidence for physically different classes of large-scale coronal EUV waves. Astron. Astrophys..

[CR113] Warmuth A., Vršnak B., Aurass H., Hanslmeier A. (2001). Evolution of two EIT/H$\upalpha$ Moreton waves. Astrophys. J. Lett..

[CR114] Warmuth A., Vršnak B., Magdalenić J., Hanslmeier A., Otruba W. (2004). A multiwavelength study of solar flare waves. I. Observations and basic properties. Astron. Astrophys..

[CR115] Warmuth A., Vršnak B., Magdalenić J., Hanslmeier A., Otruba W. (2004). A multiwavelength study of solar flare waves. II. Perturbation characteristics and physical interpretation. Astron. Astrophys..

[CR116] White S.M., Thompson B.J. (2005). High-cadence radio observations of an EIT wave. Astrophys. J. Lett..

[CR117] Wills-Davey M.J. (2006). Tracking large-scale propagating coronal wave fronts (EIT waves) using automated methods. Astrophys. J..

[CR118] Wills-Davey M.J., Attrill G.D.R. (2009). EIT waves: A changing understanding over a solar cycle. Space Sci. Rev..

[CR119] Wills-Davey M.J., DeForest C.E., Stenflo J.O. (2007). Are “EIT Waves” fast-mode MHD waves?. Astrophys. J..

[CR120] Wills-Davey M.J., Thompson B.J. (1999). Observations of a propagating disturbance in TRACE. Solar Phys..

[CR121] Wu S.T., Zheng H., Wang S., Thompson B.J., Plunkett S.P., Zhao X.P., Dryer M. (2001). Three-dimensional numerical simulation of MHD waves observed by the extreme ultraviolet imaging telescope. J. Geophys. Res..

[CR122] Wuelser J.-P., Lemen J.R., Tarbell T.D., Wolfson C.J., Cannon J.C., Carpenter B.A., Fineschi S., Gummin M.A. (2004). EUVI: The STEREO-SECCHI extreme ultraviolet imager. Telescopes and Instrumentation for Solar Astrophysics.

[CR123] Zheng R., Jiang Y., Yang J., Bi Y., Hong J., Yang B., Yang D. (2012). An extreme ultraviolet wave associated with a failed eruption observed by the solar dynamics observatory. Astron. Astrophys..

[CR124] Zhukov A.N. (2011). EIT wave observations and modeling in the STEREO era. J. Atmos. Solar-Terr. Phys..

[CR125] Žic T., Vršnak B., Temmer M., Jacobs C. (2008). Cylindrical and spherical pistons as drivers of MHD shocks. Solar Phys..

